# The experiences of sonographers with regard to report writing and communicating their findings

**DOI:** 10.4102/hsag.v27i0.2066

**Published:** 2022-11-09

**Authors:** Cassandra A. Ferreira, Barbara van Dyk, Padidi L. Mokoena

**Affiliations:** 1 Department of Medical Imaging and Radiation Sciences, Faculty of Health Sciences, University of Johannesburg, Johannesburg, South Africa

**Keywords:** ultrasound reporting, report-writing challenges, training to support sonographers, reporting structure, South Africa

## Abstract

**Background:**

Sonographers in South Africa are legally allowed to write their own reports; however, they often lack adequate training in providing a well-structured and coherent formal written report.

**Aim:**

The aim of this study was to explore and describe how sonographers in the Gauteng province experience the responsibility of report writing and to develop recommendations that could assist sonographers in the execution of their duty.

**Setting:**

Focus group discussions (FGDs) with sonographers from private and public hospitals located in Gauteng province were conducted at neutral locations that were convenient for the sonographers.

**Methods:**

A qualitative phenomenological research design was used for this study. A two-stage sampling approach was employed to recruit information-rich sonographers to partake in this study. Purposeful sampling was used to select sonographers based on their first-hand experience of report writing, followed by snowball sampling which allowed the researcher access to new participants on the recommendation of previous sonographers. Thirteen female sonographers voluntarily participated in the study, and the FGDs continued until data saturation was reached. The views and opinions of the sonographers were analysed using content analysis.

**Results:**

Key findings of this study indicated that sonographers felt unprepared to describe ultrasound findings correctly in order to provide a coherent and well-structured formal written report.

**Conclusion:**

Sonographers suggested the use of workshops or further training at higher educational institutions (HEIs) to support sonographers in their report-writing role.

**Contribution:**

The experiences identified by sonographers can assist HEIs to provide further training or workshops to support sonographers in communicating their findings effectively.

## Introduction

Globally, the role of the sonographer is to perform the ultrasound examination and record the images (American College of Radiology 2014:2–5; Australasian Society for Ultrasound in Medicine 2014:3; New Zealand Medical Radiation Technologists Board 2020:1–2; The Royal College of Radiologists 2018:4–17; United Kingdom Association of Sonographers 2008:5). In most private practices in South Africa (SA), the radiologist uses these images, together with feedback from the sonographer, to provide a written report (South African Government, Department of Health 2020:5).

However, because of a shortage of radiologists worldwide, especially in the public sector, the role of this highly operator-dependent modality has expanded significantly to include the interpretation of the examination (Australasian Society for Ultrasound in Medicine 2014:3; New Zealand Medical Radiation Technologists Board 2020:1–2) and preparation of a preliminary worksheet or report by the sonographer (Australasian Society for Ultrasound in Medicine 2014:3; Dongola et al. 2003:29, 33; Hofmann & Vikestad 2013:186; Klibanov & Hossack 2015:657; Schneider, Bloesch & Lombardo 2014:6; United Kingdom Association of Sonographers 2008:5; Yielder et al. 2014:14 & 21). In the context of SA, because of the shortage of radiologists reporting on ultrasound examinations, sonographers in the public sector compile a technical report of their findings for the referring physician, while in some private practices, sonographers write a preliminary report which is then signed off by the radiologist (Hazell, Motto & Chipeya 2015:303; Van de Venter & Ten Ham-Baloyi 2019:178; Van de Venter, Du Rand & Grobler 2016:128, 129). This requires sonographers to have extensive knowledge of anatomy, physiology and pathology in order to accurately assess their patients and compile a formal report (D’Abate & La Leggia 2017:1).

In SA, the scope of practice for sonographers has been under review since 2016 with the expectation that ‘the provision of a verbal or written report, in which normal and abnormal appearances are identified and described, while further imaging is recommended where relevant’, will formally be included in the new scope of practice in the very near future (South African Government, Department of Health 2020:5). Although report writing is not specifically mentioned in the previous scope of practice, it is implied in the Acts of Omission as stipulated by the Health Professions Council of South Africa (HPCSA), Ethical Rules of Conduct (HPCSA 2016: Booklet 2).

Around the world, sonographers have been reporting on selected ultrasound examinations with high levels of accuracy for over 30 years (Dongola et al. 2003:31; Hofmann & Vikestad 2013:188; Schneider et al. 2014:6; Van de Venter et al. 2016:128, 135–136). Proficient diagnostic ultrasound image interpretation and reporting skills can be achieved with formal report-writing training for sonographers (Dongola et al. 2003:31; Hofmann & Vikestad 2013:188; Schneider et al. 2014:6). South Africa currently offers a 4-year degree qualification with minimal report-writing training in sonography, which is covered in a single module in their third year of study over a 2-week period. Before the 4-year degree qualification, during the 2-year ultrasound degree qualification, a single lecture is offered, followed by a single test on report writing.

Some sonographers suggested that higher educational institutions (HEIs) should introduce any form of report-writing training, such as postgraduate workshops, short learning programmes or the use of templates during their 4-year degree qualification, to support sonographers in their report-writing role. The comments made by the participants are supported by Hazell et al. (2015:303) and Van de Venter and Ten Ham-Baloyi (2019:184). The 4-year degree qualification was implemented in 2016 and still does not offer support to radiographers and subsequently sonographers in their report-writing role (Hazell et al. 2015:303). The sonographers who studied during the 2-year ultrasound degree qualification have more experience in writing reports and in the field of sonography in general. Therefore, HEIs must subsequently ensure that formal report-writing training is included in ultrasound training programmes or as postgraduate workshops or short learning programmes (Necas 2018:9; Stoyles & Harrison 2006:109; United Kingdom Association of Sonographers 2008:5). This is evident from the fact that sonographers in the United Kingdom (UK), Norway and Australia are trained to write a preliminary report on ultrasound examinations of the upper abdomen (Dongola et al. 2003:29; Hofmann & Vikestad 2013:186; Schneider et al. 2014:4; Stoyles & Harrison 2006:109; United Kingdom Association of Sonographers 2008:5).

Despite sonographers being able to perform ultrasound examinations confidently, some have challenges in accurately communicating a description of the ultrasound image effectively. The aim of this study was therefore to explore and describe how sonographers in the Gauteng province experience the responsibility of report writing and to develop recommendations that could assist sonographers in the execution of this duty.

## Methodology

### Design

A qualitative phenomenological research design was employed in order to explore and describe the experiences of sonographers in Gauteng province when writing their reports. Qualitative research allowed insight into the experiences of the sonographers (Creswell 2014:4), while phenomenology allowed the researcher to explore the meaning of the lived experiences of these sonographers (Creswell 2014:14).

### Research setting

Focus group discussions (FGDs) took place in neutral environments such as quiet coffee shops or conference centres. Venues were selected for the convenience of sonographers since FGDs were scheduled for late afternoons after work.

### Study population and sample

The population for this study was all sonographers who write their own reports and are registered with the HPCSA. A two-stage sampling approach was used for this study, which employed purposeful sampling followed by snowball sampling to obtain information-rich sonographers, as there is a limited number of sonographers who write their own reports in Gauteng province (Creswell 2012:146, 206, 209). Firstly, purposeful sampling was used to select sonographers based on their first-hand experience of report writing, followed by snowball sampling, which allowed the researcher access to new participants on the recommendation of previous sonographers who had already participated in the study. Thirty-seven sonographers were purposefully invited to participate in the FGDs, of whom 23 responded and 13 volunteered and consented to taking part in the study. Qualified sonographers who met the following criteria were allowed to take part in this study: (1) compile their own reports, (2) speak English, (3) employed in the private or public sectors in Gauteng province and finally (4) registered with the HPCSA. Furthermore, participants had to be willing to share their experiences in a group setting, since data were collected with the aid of FGDs. Thirteen (*n* = 13) female sonographers volunteered and consented to participation in the study. Of these, four were employed in the private sector (*n* = 4) and nine in the public sector (*n* = 9). The sample size was determined by data saturation when no new or additional information originated from the FGDs, and as a result, a total of four FGDs were conducted. (Du-Plooy-Cilliers, Davis & Bezuidenhout 2014:136; Holloway & Wheeler 2010:146).

### Data collection

Data collection took place between October and November 2018. Close attention was paid to the sonographers’ facial expressions and body language, and for this reason, field notes were compiled by the researcher during each FGD. During the FGDs, a semistructured interview process was utilised to gain insight into their experiences. The central question used to guide each discussion was: ‘Tell me about your experiences of writing your own report’, which was followed by additional probing questions and paraphrasing emanating from the sonographers’ responses. All FGDs were conducted in English and recorded on an audio recorder with the permission of each sonographer. Each FGD lasted for about an hour. Reflective notes were made before and after each FGD. The researcher then listened to each audio-recording repeatedly before transcribing the FGDs from the audio recorder.

Focus group discussions were utilised for this study in order for the researcher to obtain valuable information by using group interactions to explore the experiences of the sonographers regarding report writing (Bradbury-Jones, Sambrook & Irvine 2009:665; Creswell 2012:218; Creswell 2014:190). Group interviews are beneficial in phenomenological studies since they stimulate discussion and open up new perspectives (Bradbury-Jones et al. 2009:665). The use of focus groups subsequently allowed the researcher to obtain a wide range of data rapidly and provided sonographers with an opportunity to shed light on issues of importance to them, which gave more distinction to their perceptions about the research topic (Bradbury-Jones et al. 2009:666).

### Data analysis

Data analysis commenced by the researcher listening to the audiotapes repeatedly, after which the interviews were transcribed verbatim. This allowed the researcher to obtain the general sense of the experiences of sonographers’ report writing. Field notes of nonverbal behaviours of the sonographers were compiled during the FGDs and were used during this phase for triangulation of results (Creswell 2014:197). The transcripts were subsequently used to formulate a general impression of the overall meaning of the data (Creswell 2012:236, 2014:197). Certain words came to the fore, and as the data were continuously interrogated, broad themes were recognised with subcategories under each theme. The data were subsequently coded according to ‘meaning units’ while related codes were grouped into categories which each describe a different aspect of the raw data. Themes were then developed to express the underlying meaning in each category (Erlingsson & Bryciewicz 2017:2, 4–5). A consensus meeting was held between the researcher and an independent coder who has extensive experience in coding qualitative research to discuss the themes and categories identified by the researcher.

### Measures of trustworthiness

To ensure the trustworthiness of the research findings, the principles from Lincoln and Guba’s model (1985:301–327), as described by Murphy and Yielder (2010:65), were employed in this study. The following trustworthiness measures were therefore employed by the researcher: credibility; transferability; dependability and confirmability.

**Credibility** was ensured by triangulation of the data obtained from the FGDs together with the researcher’s reflective and field notes. Member checking was achieved by summarising the participant’s viewpoints and asking them to confirm, via e-mail, if the researcher’s understanding of their viewpoints was an accurate reflection of their intention. Prolonged engagement was achieved by building a rapport with the sonographers in order to develop a trust relationship with them.

**Transferability** was ensured by providing a detailed step-by-step description of the research process, together with a detailed description of demographic information of the sonographers in order to allow other researchers to repeat or replicate the study in another setting. Likewise, **dependability** was ensured by providing a detailed description of data collection, analysis and interpretation and a dense description of the study design (Lincoln & Guba 1989:317). Lastly, **confirmability** was ensured by triangulation of data, an audit trail and bracketing (through reflexivity). Reflexivity is the consciousness of the biases, values and experiences that the researcher brings into the research study. A conscious decision was thus made by the researcher to monitor actions that could introduce bias and ultimately threaten the credibility of the study (Holloway & Wheeler 2010:8). All these measures ensured that the voice of each participant was clearly heard throughout the process (Holloway & Wheeler 2010:303; Murphy & Yielder 2010:65).

### Ethical considerations

Ethical clearance was obtained from the University of Johannesburg’s Faculty of Health Science Research Ethics Committee (reference number: REC-01-80-2018) and Higher Degrees Committee of the Faculty of Health Sciences (reference number: HDC-01-52-2018). Consent for participation and the recording of interviews was obtained from all participants before data collection commenced. Participants were free to leave the focus group sessions at any time without any consequences. None of the participants left any of the focus group sessions. Participants were ensured that their privacy would be protected at all costs and that the data would be presented anonymously through the use of pseudonyms.

## Results

The most relevant and meaningful quotes relating to the themes and subthemes are presented below with verbatim comments of the participants (P) presented in quotes.

### Theme 1: The challenges sonographers face during the report-writing process

Following data analysis, two subthemes were identified: the continuous challenge faced to describe the ultrasound findings accurately and the fear of making a misdiagnosis.

#### Subtheme 1: The continuous challenge faced to describe the ultrasound findings accurately

A couple of the participants expressed that they have a lack of confidence to provide an accurate description of their findings:

‘So you might know what’s going on, on the ultrasound scan you did, but you won’t be able to say it nicely.’ (FGD 1, P4, 17 Oct. 2018).

‘Whatever that you’ve seen and you don’t know what, you can describe even if you don’t know the exact pathology of what you’ve seen.’ (FGD 3, P1, 17 Nov. 2018)

The participants asserted that although they have all this knowledge of ultrasound and confidence in their scanning abilities, they feel lost when they have to put pen to paper to describe their findings. Some expressed that while they have no problem communicating their findings verbally, they have difficulties with how to word them in a formal report.

‘The wording. I don’t know what’s happening.’ (FGD 1, P1, 17 Oct. 2018)

‘Well, as a newly qualified, it’s very stressful, um, from phrasing your sentences to trying to get, you know, the most accurate, you know, description. So for me it’s very stressful, ya.’ (FGD 2, P1, 31 Oct. 2018)

All participants agreed that their aim is to describe their findings in a way which is accurately translating what they have seen during the scan. The report ultimately serves as a guide for the referring physician towards the diagnosis and appropriate treatment.

‘And because your report is not a diagnosis but a description, it’s a description of a finding.’ (FGD 2, P2, 31 Oct. 2018)

The wording of the ultrasound report hence plays an important part in guiding the clinician towards a diagnosis, and sonographers need to word, phrase and structure their reports so that they provide a clear description of their findings.

#### Subtheme 2: The fear of misdiagnosis

To misdiagnose or give incorrect information on a particular illness or condition is one of the biggest fears expressed by sonographers in this study. Not only does it impact the patient outcome in a negative way, it also negatively reflects the capability of the sonographer. Sonographers do not intentionally miss pathology; however, they can sometimes misinterpret their findings and thus report on them incorrectly (Van de Venter & Ten Ham-Baloyi 2019:178; Van de Venter et al. 2016:131).

‘I think on my side, I do worry a lot because in case I have this question, what if I misdiagnose the patient and they give the patient the wrong treatment?’ (FGD 1, P2, 17 Oct. 2018)

Sonographers do not purposefully misdiagnose a patient. One of the participants was visibly upset when she pointed out in the statement below that referring physicians tend to take the sonographer’s word as final. These situations can create hesitancy on the sonographer’s part as they may fear that the referring physician could misconstrue their report and opinion as a final diagnosis (Van de Venter et al. 2016:131):

‘They shouldn’t be taking your word as the final word, that this is definitely what it is, but they tend to do that. So for me that is very challenging. And I think it’s wrong, but that’s what it is, they just take our word for it. So as a sonographer, honestly, one has to be as accurate as possible, if I can put it that way.’ (FGD 3, Deedee, 17 Nov. 2018)

Schneider et al. (2014:6) state that: ‘Sonographers’ findings are not intended to be more than a tool to communicate to the radiologist and are not designed to convey a diagnosis to referring clinicians’. However, studies show that sonographers do convey accurate findings to the referring clinicians (Dongola et al. 2003:31; Hofmann & Vikestad 2013:188; Schneider et al. 2014:6). The above statement from this participant emphasises the need for skills development to convey an accurate report to referring clinicians.

### Theme 2: The need for skills development as a way forward for sonographers

This theme emanated in response to the challenges as a necessary suggestion for the development and optimisation of sonographers’ skills.

Many of the participants hold the opinion that there is a need for skills development with regard to writing more coherent and formal reports. Participants agreed enthusiastically that either more in-depth lectures or workshops would benefit future sonographers. Most participants felt that aspects of report writing which need more practice and guidance include wording reports correctly, what to include in the report and how to structure.

‘But I feel like there was no proper or enough time where they sit down with you and tell you this is how you break down your report and this is how you put it down.’ (FGD 1, P2, 17 Oct. 2018)

‘Ya, an in-depth, ‘cause I mean, now they spread it [training] over four years, so maybe it could be looked at in their third or fourth year of training that they have, like, an in-depth module on report writing. ‘Cause the phrasing is important, you know, that type of thing.’ (FGD2, P2, 31 Oct. 2018)

The report needs to be structured in a way which is comprehensible to both the author and the reader, so that specific phrases and words are understood clearly (Lee & Whitehead 2016:189; Wilcox 2006:33).

‘Whatever you’ve seen, the pathology that you’ve seen, because we’ve got pictures that we look at, it’s always easier to remember what you’ve seen and then write out whatever that it is that you want to report on.’ (FGD 3, P1, 17 Nov. 2018)

#### Subtheme 1: Professional growth and skills development through further education, training and workshops

It is evident from the above and below comments that there is a need for further training and development of report-writing skills. This training will not only benefit sonographers in writing well-structured and coherent formal reports, but it will also widen their knowledge and enable them to reason in the way radiologists and doctors do.

‘[…I]f we can be given a range of time, have specific lectures where we, like, sit down and try to break it down into proper way of writing reports, not like a sonographic evaluation in an exam. I think it will really help.’ (FGD 1, P2, 17 Oct. 2018)

Most participants held the opinion that their training institutions did not place sufficient emphasis on developing their reporting skills and that the majority of their experience was obtained from their senior colleagues or radiologists. They specifically feel that there was a lack of training on how to structure formal reports, which resulted in them struggling with the correct phrasing to use. Furthermore, they expressed the view that more in-depth training or workshops could be highly beneficial for future and current sonographers to aid them in their report-writing role.

‘I think the institutions that are teaching ultrasound, like universities, I think they need to put it into consideration on teaching the students how to write the reports. Proper lectures, not like to learn from, just from the sonographers, because sometimes it’s very busy.’ (FGD 1, P2, 17 Oct. 2018)

‘A more in-detail type of lecture at university. Not just like a once-off, this is what you say, and then the learners still come out of there not knowing left from right.’ (FGD 2, P2, 31 Oct. 2018)

#### Subtheme 2: Adopting a structured reporting format

Since constructing formal reports seems to be lacking as a skill, especially with young and newly qualified sonographers, a few of the more experienced participants suggested the use of templates as a solution to the problem.

‘I know there’s some institutions that are now using templates for certain things, so maybe the universities should make use of a template to at least formulate something where they can use that for teaching students on how to write the report. At least it’s something, so when the student goes out into the workplace they have something, not having to lean on – not that that’s wrong, but know that you at least come with knowledge.’ (FGD 2, P2, 31 Oct. 2018)

‘Having said that, you have a template; the template is just the guide, you know, because no two cases are going to be exactly the same.’ (FGD 2, P2, 31 Oct. 2018)

‘So if that template’s been taught somehow, maybe that could be a guide or something to help them.’ (FGD 2, P2, 31 Oct. 2018)

The quote below from one of the participants speaks of the aspects of report writing with a template when abnormalities are seen:

‘So speaking from a different aspect with regard to report writing, there are templates that make it a bit easier to write reports which we use in our department but we still - when we find abnormalities, we use more of a descriptive method in describing things than outright stating what we see.’ (FGD 4, P2, 20 Nov. 2018)

The aforementioned quote shows how she is aware of the benefits of a template but also knows that further information may still be required to provide an accurate and comprehensive report.

## Discussion

The main findings of this study revealed the challenges sonographers face while writing their own reports. This study also indicated that sonographers felt the need for further education and training in order to improve their knowledge and report-writing skills. Sonographers who completed the 2-year degree qualification had more experience in writing reports as opposed to sonographers who completed the 4-year degree qualification.

### The challenges sonographers face during the report-writing process

The data collected from this study indicates high levels of uncertainty among sonographers on how to construct formal reports. Data and the literature further indicate that although sonographers are capable of providing an accurate interpretation of ultrasound scans, wording their findings in a coherent and concise manner is at times daunting (Hazell et al. 2015:303; Neep et al. 2014:73; Smith, Traise & Cook 2009:7). Subsequently, the essence of the report may be lost because the report becoming more of a description as opposed to the provision of differential diagnoses. In keeping with the literature, some participants additionally felt that they lacked an in-depth knowledge of pathologies or clinical presentation of diseases in order to provide differential diagnoses in their reports (Hazell et al. 2015:303; Neep et al. 2014:73; Smith et al. 2009:7).

The ultrasound report is the main tool used to communicate the interpretation of the ultrasound examination to the referring physicians (Lee & Whitehead 2016:186; Wallis et al. 2013:1146; Zulkarnain, Crofts & Meziane 2015:508). It is, furthermore, a medicolegal document that is the intellectual property of the imaging department and should provide adequate information regarding the ultrasound examination, while highlighting important findings to answer the clinical questions (Boland, Guimaraes & Mueller 2008:1326; Wallis et al. 2013:1146).

Since the ultrasound report plays a vital role in reaching a diagnosis, it should be written in a clear and concise way to guide the referring physician towards a conclusion (Ridley 2002:366; Zulkarnain & Meziane 2019:1; Zulkarnain et al. 2015:508). Referring physicians thus rely heavily on the radiology report, as it often determines the treatment of the patient (Boland et al. 2008:1326). It is important to understand that sonographers are providing an opinion based on the ultrasound findings and occasionally are advising on further investigations and follow-up scans, while taking the relevant clinical history into account (Ridley 2002:367–369).

It is of no benefit to the patient if no report or opinion is received on a diagnostic test, since this may translate into the mismanagement or even misdiagnosis of the patient (Van de Venter & Ten Ham-Baloyi 2019:178). Van de Venter et al. (2016:133, 134–135) and Smith et al. (2009:8) argue that when sonographers collaborate with referring physicians and discuss their findings, a good working relationship is created which holds positive benefits for the patient.

As mentioned earlier, sonographers have the knowledge and are confident in detecting abnormalities; however, some sonographers have difficulty translating their findings into words and lack the vocabulary to provide an accurate comment on their findings (Hazell et al. 2015:303; Neep et al. 2014:73; Smith et al. 2009:7). Thus, the risk of misdiagnoses is reduced when knowledge is applied correctly and linked to the patient’s clinical history (Van de Venter & Ten Ham-Baloyi 2019:184; Van de Venter et al. 2016:134–135; Williams et al. 2018:14).

### The need for skills development and change as a way forward for sonographer

Supported by the College of Radiographers, sonographers in the UK have been reporting for many years, with independent reporting becoming more common (Stoyles & Harrison 2006:109). Formal training on how to describe ultrasound findings has been advised to improve the professionalism, confidence and communication skills of sonographers (Hazell et al. 2015:307; McGregor et al. 2009:317; Neep et al. 2014:74; Schneider et al. 2014:6). However, little to no formal training in report-writing technique and style is provided for sonographers at the university level (McGregor et al. 2009:317; Wallis & McCoubrie 2011:1051; Wallis et al. 2013:1147).

Short learning programmes or workshops to address the skills required by sonographers in order to assist them in their report-writing role could be offered by HEIs. Formal training on how to describe ultrasound findings is advised to improve the professionalism and communication skills of sonographers (Hazell et al. 2015:307; Neep et al. 2014:74; Schneider et al. 2014:6).

Workshops or written material, furthermore, provide guidelines on reporting style, report content and pitfalls to be avoided. The American College of Radiology (2014:2–5), the Royal College of Radiologists (2018:4–17) and the British Medical Ultrasound Society (BMUS 2015:40–41) are among the professional organisations who issued guidelines and reporting standards which can be used to formulate reporting templates to further assist sonographers in their report-writing role. Higher education institutions in South Africa could refer to these guidelines in an effort to develop workshops in aid of better practice.

Image interpretation coursework for radiography at a postgraduate level is already available in some countries, including Denmark, the UK and Australia (Neep et al. 2014:70; Williams et al. 2018:15; Wuni, Courtier & Kelly 2020:123). These countries have paved the way for radiographers in general and sonographers in particular to function as reporting consultants in their respective healthcare systems (Neep et al. 2014:70; Williams et al. 2018:15; Wuni et al. 2020:123).

It has been proved that with adequate training, sonographers can achieve high levels of accuracy and agreement with reports issued by radiologists (Dongola et al. 2003:31; Hofmann & Vikestad 2013:188; Schneider et al. 2014:6). However, without adequate preparation for a reporting role, the phrasing of a report may deliver an unclear message to the reader. Thus, the structure of the report needs to be comprehensible to provide a clear understanding of specific words and phrases (Lee & Whitehead 2016:189; Wilcox 2006:33). Schneider et al. (2014:6) also suggest that formal training in report writing would improve the accuracy with which the ultrasound findings are described, thus assisting the referring physicians in the interpretation of the findings (Neep et al. 2014:70 & 74; Williams 2006:16; Wuni et al. 2020:123). It is evident that additional training for sonographers will positively impact their ability to accurately describe their findings, as has been proved true for diagnostic radiographers who received postgraduate training. Hence, HEIs in South Africa could learn from the experience of their peers across the globe by reviewing and adapting existing curricula to better prepare sonography students for advanced professional roles (Stevens & Thompson 2018:48; Wuni et al. 2020:123). Furthermore, Wuni et al. (2020:123) and Williams (2006:15) suggest that the radiography curriculum should be reviewed to ensure that undergraduate curricula include components of image interpretation and clinical reporting.

Studies from the UK, Belgium and the United States have discussed the use of structured reporting as an aid to radiologists and reporting sonographers to minimise variations in reporting styles and standardise terminologies (Bosmans et al. 2012:296; Zulkarnain & Meziane 2019:1; Zulkarnain et al. 2015:508). There is, furthermore, an assumption that the accuracy of reports has improved with the use of structured reporting templates (Bosmans et al. 2012:297; Zulkarnain et al. 2015:508).

However, to fulfil the need expressed by qualified sonographers, components of report writing and advanced pathology will need to be elaborated upon to meet expectations and ensure better practice standards (Neep et al. 2014:70; Williams et al. 2018:15; Wuni et al. 2020:123).

## Limitations and recommendations

A limitation of this study was the small sample size, which might have resulted in the omission of important insights from the perspective of independent sonographers. The small sample, however, is attributed to the fact that there is a limited number of sonographers who write their own reports in Gauteng province. It is therefore recommended that sonographers from other provinces should be considered to potentially provide additional insights.

## Conclusion

This study explored and described the experiences of sonographers in Gauteng province regarding report writing. The findings indicated that the challenges faced by sonographers could be addressed through workshops or further training by HEIs to support sonographers in their report-writing role. Healthcare education, in general, can benefit from a more clinically based learning style (Gibbs 2011:31) and ongoing education to support sonographers in their report-writing roles (McGregor et al. 2009:317; Williams 2006:15; Wuni et al. 2020:123).
